# NHash: Randomized N-Gram Hashing for Distributed Generation of Validatable Unique Study Identifiers in Multicenter Research

**DOI:** 10.2196/medinform.4959

**Published:** 2015-11-10

**Authors:** Guo-Qiang Zhang, Shiqiang Tao, Guangming Xing, Jeno Mozes, Bilal Zonjy, Samden D Lhatoo, Licong Cui

**Affiliations:** ^1^ Institute of Biomedical Informatics University of Kentucky Lexington, KY United States; ^2^ Center for SUDEP Research (NINDS-funded Center Without Walls for Collaborative Research in the Epilepsies) Cleveland, OH United States; ^3^ Department of Computer Science Western Kentucky University Bowling Green, KY United States; ^4^ Department of Neurology Case Western Reserve University Cleveland, OH United States

**Keywords:** cryptographic hash function, multi-center study, study identifiers, health information management, data integration, patient cohort identification, search interface

## Abstract

**Background:**

A unique study identifier serves as a key for linking research data about a study subject without revealing protected health information in the identifier. While sufficient for single-site and limited-scale studies, the use of common unique study identifiers has several drawbacks for large multicenter studies, where thousands of research participants may be recruited from multiple sites. An important property of study identifiers is error tolerance (or validatable), in that inadvertent editing mistakes during their transmission and use will most likely result in invalid study identifiers.

**Objective:**

This paper introduces a novel method called "Randomized N-gram Hashing (NHash)," for generating unique study identifiers in a distributed and validatable fashion, in multicenter research. NHash has a unique set of properties: (1) it is a pseudonym serving the purpose of linking research data about a study participant for research purposes; (2) it can be generated automatically in a completely distributed fashion with virtually no risk for identifier collision; (3) it incorporates a set of cryptographic hash functions based on N-grams, with a combination of additional encryption techniques such as a shift cipher; (d) it is validatable (error tolerant) in the sense that inadvertent edit errors will mostly result in invalid identifiers.

**Methods:**

NHash consists of 2 phases. First, an intermediate string using randomized N-gram hashing is generated. This string consists of a collection of N-gram hashes *f*_1_, *f*_2_, ..., *f*_*k*_. The input for each function *f*_*i*_ has 3 components: a random number *r*, an integer *n*, and input data *m*. The result, *f*_*i*_(*r*, *n*, *m*), is an n-gram of *m* with a starting position *s*, which is computed as (*r* mod |m|), where |m| represents the length of *m*. The output for Step 1 is the concatenation of the sequence *f*_1_(*r*_1_, *n*_1_, *m*_1_), *f*_2_(*r*_2_, *n*_2_, *m*_2_), ..., *f*_*k*_(*r*_*k*_, *n*_*k*_, *m*_*k*_). In the second phase, the intermediate string generated in Phase 1 is encrypted using techniques such as shift cipher. The result of the encryption, concatenated with the random number r, is the final NHash study identifier.

**Results:**

We performed experiments using a large synthesized dataset comparing NHash with random strings, and demonstrated neglegible probability for collision. We implemented NHash for the Center for SUDEP Research (CSR), a National Institute for Neurological Disorders and Stroke-funded Center Without Walls for Collaborative Research in the Epilepsies. This multicenter collaboration involves 14 institutions across the United States and Europe, bringing together extensive and diverse expertise to understand sudden unexpected death in epilepsy patients (SUDEP).

**Conclusions:**

The CSR Data Repository has successfully used NHash to link deidentified multimodal clinical data collected in participating CSR institutions, meeting all desired objectives of NHash.

##  Introduction

Unique study identifiers, or pseudonyms, are alphanumeric codes used in clinical and other research studies to uniquely identify a study participant without revealing in the identifiers any Personal Health Information (PHI) [1], such as name, full date of birth (DOB), and medical record number (MRN) [2]. For a fictional study participant, Aaron Skotnica, with DOB 08/13/1956 and MRN 07172485, the unique study identifier could be a number such as 57, representing the 57th enrolled study subject. Or it could be a randomly generated number, such as 28262. However, a large number of more sophisticated mechanisms for generating unique study identifiers do exist. A separate codebook, stored and managed in a secure environment, links the unique study identifier to the actual research participant. Electronic data files with unique study identifiers, even de-identified, must be stored in a secure and protected manner, with access granted only to authorized study personnel approved by institutional review boards.

While sufficient for single-site and limited scale studies, the use of common unique study identifiers has several drawbacks for large multicenter studies, where thousands of research participants may be recruited from multiple sites. These drawbacks include (1) the need for coordination so that different sites use distinct blocks of non-overlapping codes for unique study identifier to avoid collision (ie, the same identifier is generated for distinct subjects), (2) difficulty validating if a piece of alphanumeric code is a legitimate unique study identifier or not (eg, if the unique study identifier 57 is inadvertently transposed to 75, the result could be another valid unique study identifier, but for a different study participant), and (3) the possibility that aggregated site-specific information could easily be derived, which may be undesirable if distinct code segments are used for distinct sites, after merging study data in a central study repository.

This paper introduces a novel method called Randomized N-gram Hashing (NHash), for generating unique study identifiers for multicenter research. A study identifier generated using NHash has a unique set of properties: (1) as a unique study identifier, it is a pseudonym serving the purpose of linking research data about a study subject for research purposes, (2) it can be generated automatically in a completely distributed and decentralized fashion, yet allowing data integration with virtually no risk for identifier collision, (3) it incorporates a set of cryptographic hash functions based on N-grams for its generation, which can be further encrypted if desired, using encryption techniques such as shift-encryption, and (4) it is validatable in the sense that inadvertent edit errors on NHash identifiers, during their use, will almost always result in invalid identifiers. Furthermore, it is straightforward to validate if the codebook linking study subject and the associated NHash identifier contains errors, simply by regenerating the NHash identifier using the random number with patient information to generate the decryption keys.

**Figure 1:**
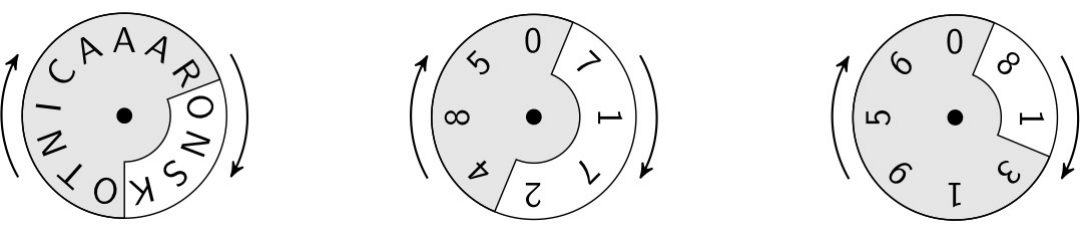
An intermediate string, ONSK717281, is generated using the concatenation of 4-gram ONSK from name, 4-gram 7172 from MRN, and 2-gram 81 from DOB, with N-gram hash functions based on a random number 783305.

For the same fictional study participant, Aaron Skotnica, an NHash identifier is TSXP606170783305. This is achieved by first obtaining an intermediate string, ONSK717281, based on a randomly generated number, 783305. The intermediate string is obtained as the concatenation of the 4-gram of first-name-last-name starting from position x, the 4-gram of MRN starting from position y, and the 2-gram of DOB starting from position z (see Figure 1), where x=3 (783305 mod 13), y=1 (783305 mod 8), and z=1 (783305 mod 8).

The intermediate string, ONSK717281, is then further encrypted to obtain TSXP606170 by shifting each letter in the alphabetic order by 5 (239 mod 26) and each digit by 9 (239 mod 10). The number 239 is calculated using Cantor paring function: ½(k_1_+k_2_)(k_1_+k_2_+1)+k_2_. Here k_1_=13 (length of the participant’s full name) and k_2_=8 (the participant’s birth month). Concatenating TSXP606170 with the random number, 783305, obtains TSXP606170783305 as the final NHash identifier.

We have implemented NHash for the Center for SUDEP Research (CSR), a National Institute for Neurological Disorders and Stroke (NINDS)–funded Center Without Walls for Collaborative Research in the Epilepsies. The CSR Data Repository uses NHash-identifiers to link de-identified multimodal clinical data collected in participating institutions. Since the official launching of CSR in December 2014, 341 study subjects have been enrolled in the Sudden Unexpected Death in Epilepsy Patients (SUDEP) study, with nearly 7TB of EEG signals linked using NHash-identifiers.

### Background

#### Protected Health Information

The broader context for effort in the creation of unique study identifiers is the Privacy Rule of the Health Insurance Portability and Accountability Act of 1996 (HIPAA). HIPPA creates a set of requirements and restrictions for the handling of PHI, which is defined as a subset of individually identifiable health information created or received by a health care provider in a variety of forms, including identifiers that could be used to uniquely determine the individual. PHI can come from demographic information, medical history, test and laboratory results, insurance information, and other data that are collected by a health care professional during the delivery of care.

De-identification is a process in which PHI elements are eliminated or manipulated with the purpose of hindering the possibility of revealing PHI contained in the original dataset. This involves removing all identifying data to create unlinkable data. One method of de-identification under HIPPA (called the Safe Harbor Method) used for the current study is when data have been stripped of 18 common identifiers found in patient names, geographic data, all elements of dates, telephone numbers, fax numbers, email addresses, social security numbers, or medical record numbers.

#### Unique Study Identifiers

A unique study identifier serves the purpose of linking research data about a study subject without revealing PHI in the identifier. With unique study identifiers, de-identified data can be coded with the possibility of linking to the original, fully identified dataset kept by an honest broker or authorized study personnel. The methods for the creation of unique study identifiers range from manual, incremental counts, to completely randomized and encrypted. For example, for the National Database for Autism Research [3], a centralized method for generating global unique identifiers to link collections of research data and specimens is used. For generating such types of global unique identifiers, a Web service is provided for an investigator to input identifying information about a participant into a client application. This information is then encrypted and sent to a server application, returning a generated global unique identifier to the original requester.

#### Center for SUDEP Research

Epilepsy is the most common serious neurological disorder, affecting 65 million persons worldwide; 200,000 new cases of epilepsy are diagnosed in the United States each year [4]. A third of epilepsy patients fail medical treatment and continue to have seizures [5,6]. SUDEP is the leading mode of epilepsy-related death and is most common in patients with intractable, frequent, and continuing seizures.

The Center for SUDEP Research (CSR) is a National Institute for Neurological Disorders and Stroke (NINDS)–funded Center Without Walls for Collaborative Research in the Epilepsies. This milestone-driven collaboration is composed of researchers from 14 institutions across the United States and Europe and brings together extensive and diverse expertise to SUDEP.

To address the challenges of data integration and data access from multiple Epilepsy Monitoring Units (EMUs), we developed the Multi-Modality Epilepsy Data Capture and Integration System (MEDCIS) [7] that combines retrospective clinical free-text processing using natural language processing (NLP), prospective structured data capture using an ontology-driven interface, and interfaces for cohort search and signal visualization, all in a single integrated environment. A dedicated Epilepsy and Seizure Ontology [8] has been used to streamline the user interfaces, enhance usability, and enable mappings across distributed databases so that federated queries can be executed.

The data capturing component of MEDCIS is called OPIC: Ontology-driven Patient Information Capture [9]. Among the 14 participating institutions, each of the 9 clinical sites will deploy an OPIC instance in their hospital EMUs. A decentralized or distributed study identifier generation method is therefore needed for CSR because (1) there is no centralized management of the codebook to link study identifiers to unique patients, and (2) a global unique identifier generation service involves extra book-keeping and the use of Web services outside the hospital firewall environment.

## Methods

Our NHash algorithm generates a unique study identifier for each participant taking, for example, name, MRN, and DOB as input, using randomized N-gram Hashing.

### N-Grams

In the fields of computational linguistics, an N-gram is a contiguous sequence of *N* items from a given sequence of text or speech. The items are basic units of code appropriately defined for each application: it can be syllables, letters, words, or base pairs in bioinformatics. An N-gram of size 1 is referred to as a “unigram,” size 2 is a “bigram,” and size 3 is a “trigram.” For example, ONSK is a 4-gram from AARONSKOTNICA.

### Cryptographic Hashing

A cryptographic hash function is a hash function that is considered practically impossible to invert, that is, to recreate the input data from its hash (output) value. These “one-way” hash functions are basic building blocks of modern cryptography. A good cryptographic hash function should have four main properties: (1) it is easy to compute the hash value for any given input data, (2) it is infeasible to generate the input data from merely the hash value, (3) it is infeasible to modify an input data without changing the hash value, and (4) it is infeasible to find two different inputs with the same hash value (false identity).

#### NHash: Randomized N-gram Hashing for Generating Study Identifiers

NHash consists of two phases. First, an intermediate string using randomized N-gram hashing is generated. This string consists of a collection of N-gram hashes *f*_*1*_ *, f*_*2*_, … , *f*_*k*_. The input for each function *f*_*i*_ has three components: a random number *r*, an integer *n*, and input data *m*. The result, *f*_*i*_(*r, n, m*), is an *n-*gram of *m* with a starting position *s*, which is computed as *(r* mod |*m*|*)*, where |*m*| represents the length of *m*. The output for Step 1 is the concatenation of the sequence *f*_*1*_ *(r*_*1*_ *, n*_*1*_ *,m*_*1*_ *), f*_*2*_ *(r*_*2*_ *, n*_*2*_ *, m*_*2*_ *), … , f*_*k*_ *(r*_*k*_ *, n*_*k*_ *, m*_*k*_ *)*.

In the second phase, the intermediate string generated in the first phase is encrypted using techniques such as shift-cipher in order not to reveal actual letters in patient names nor digits in DOB or MRN. The result of the encryption, concatenated with the random number *r*, is the final NHash study identifier.

For the CSR clinical and research data platform, we take *k*=3, *r*=*r*_*1*_=*r*_*2*_=*r*_*3*_, and *m*_1_ to be the first-name last-name string, *m*_2_ to be the MRN number, and *m*_3_ to be the digital version of DOB. Further, we take *n*_*1*_=4, *n*_*2*_=4, and *n*_*3*_=2, that is, a 4-gram of name, a 4-gram of MRN, and a 2-gram of DOB. The starting positions for these N-grams are determined by *r* modular the length of *m*_*1*_, *m*_*2*_ *,* and *m*_3_, respectively. Table 1 contains an example illustrating the notion of N-gram hash functions based on the fictional study participant, Aaron Skotnica (also shown in Figure 1).

**Table 1:** Example of the N-gram hash functions used for CSR, generating the final output TSXP606170783305.

*i*	*r*_*i*_	*n*_*i*_	*m*_*i*_	|*m*_*i*_|	*s*_*i*_ =(*r*_*i*_ mod |*m*_*i*_|)	*f*_*i*_(*r*_*i*_ *, n*_*i,*_ *m*_*i*_)	Shift-cipher
1	783305	4	AARONSKOTNICA	13	3	ONSK	TSXP
2	783305	4	07172485	8	1	7172	6061
3	783305	2	08131956	8	1	81	70

**Figure 2:**
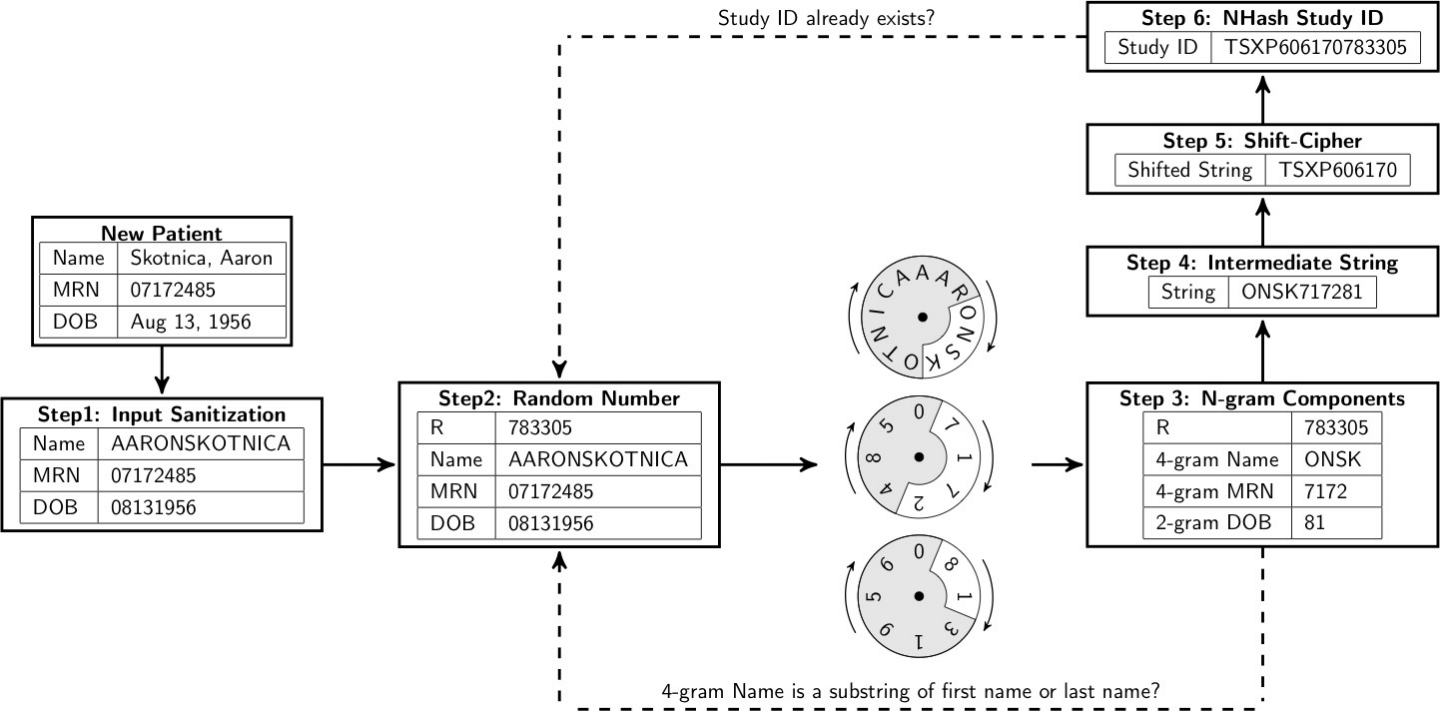
Illustrative diagram for the algorithm to generate NHash study ID.

**Figure 3:**
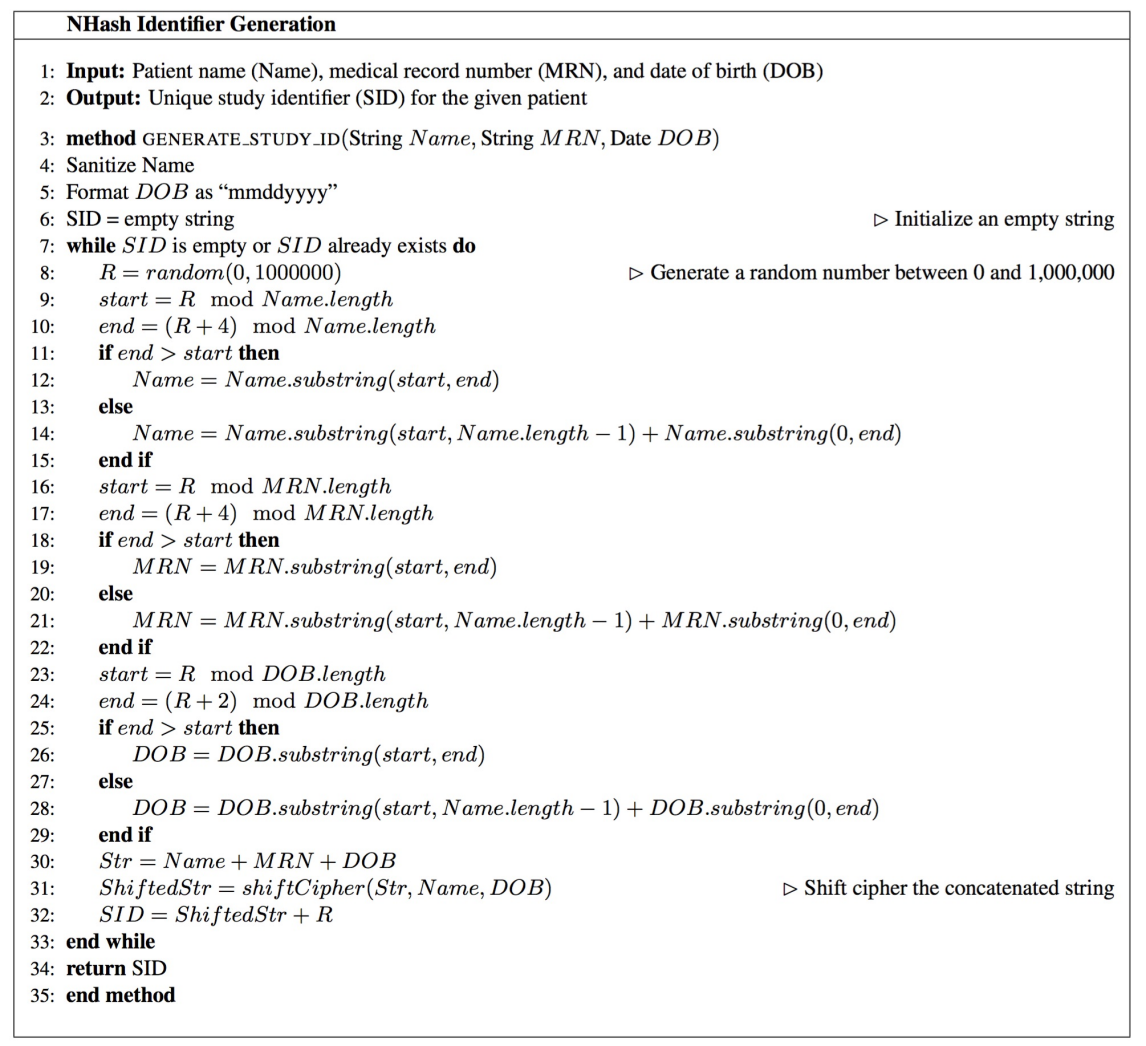
NHash algorithm to generate unique study identifier given patient demographic information.

Figure 2 illustrates the CSR NHash study identifier generation in 6 steps. Given a study participant with required information on name, MRN, and DOB, the input is sanitized first by removing possible punctuation in the name and formatting the date of birth in the format “mmddyyyy.” A random number R between 0 and 1,000,000 is generated next. In the third step, a 4-gram from name (name component), a 4-gram of medical record number (MRN component), and a 2-gram from date of birth (DOB component) are extracted from the sanitized input. The starting position for each N-gram is calculated using modular arithmetic. In Step 4, we concatenate these 3 components. In Step 5, we encrypt the concatenated string from Step 4 using such means as shift-cipher. In Step 6, we concatenate the encrypted string and the random number and output it as the unique study identifier. In the same step, we also invoke a duplication check to see if an NHash study ID already exists in the local instance, before the generated study ID is finally put into real use. Step 2 is repeated if a local collision is detected. These six steps are captured more formally as an algorithm in pseudo code (Figure 3).

### Integration With Ontology-Driven Patient Information Capturing System for Epilepsy

The NHash study identifier generator is an integrated component of OPIC. OPIC captures patient discharge summary reports in EMUs and has a semi-automated built-in de-identification process making clinical data suitable for research for properly enrolled patients. Since one patient can make multiple clinical visits to the EMU and each visit will produce a discharge summary report, the patient study identifier itself is not sufficient enough for naming discharge summary reports directly. To address this phenomenon, we supplement the NHash identifier with an additional 2-digit number and postfix it to the NHash identifier. This way, a discharge summary report is uniquely identified by the NHash identifier plus a 2-digit number postfix. For example, TSXP60617078330501.pdf, TSXP60617078330502.pdf, TSXP60617078330503.pdf, TSXP60617078330504.pdf, if they exist in OPIC, would be the names of discharge summary reports for the first four visits of fictional study subject, Aaron Skotnica.

### Linking CSR Multimodal and Multicenter Clinical Research Data

**Figure 4:**
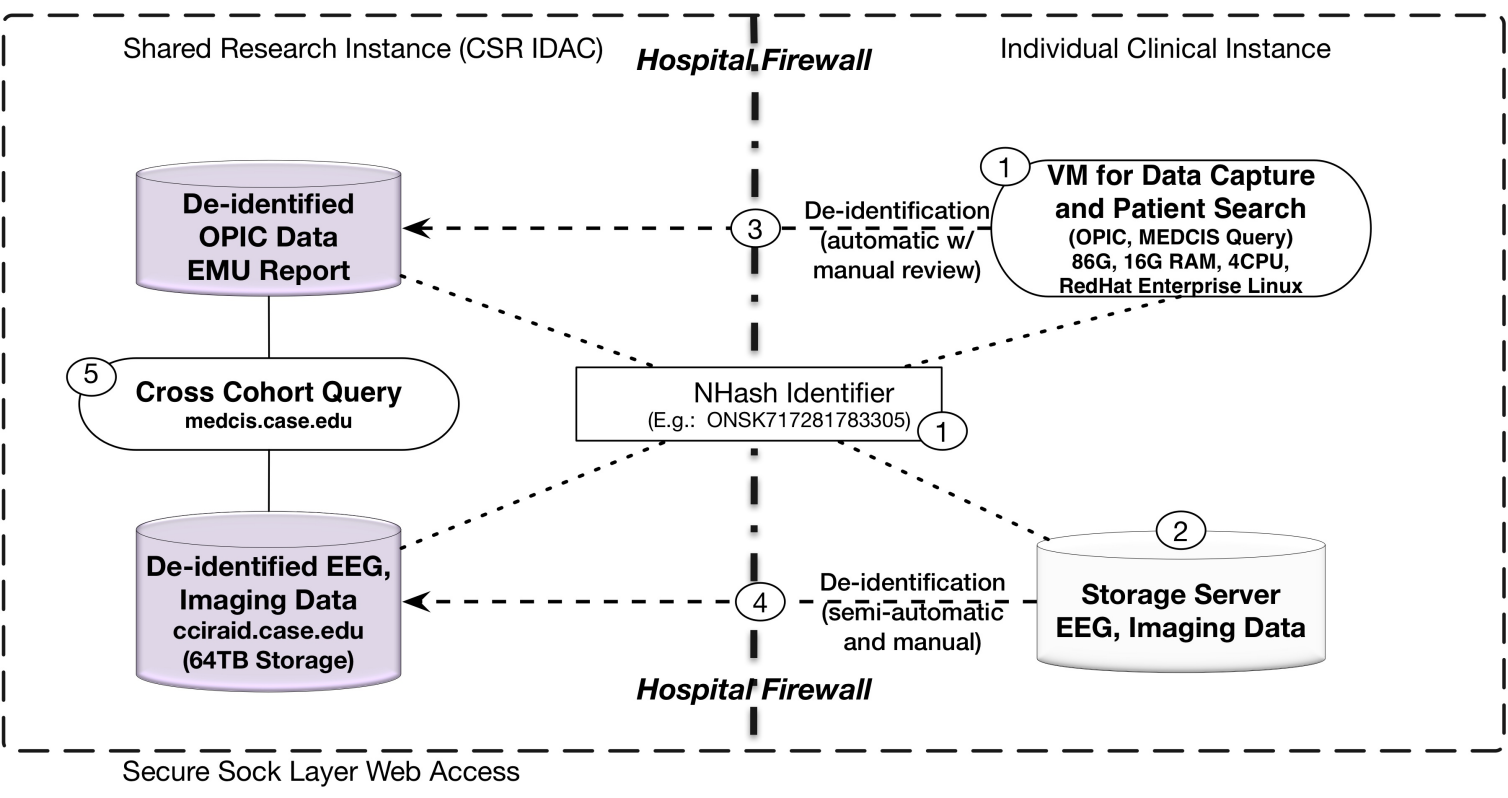
CSR data flow and record linking enabled by NHash identifier.

Figure 4 is the CSR data flow architecture for linking multimodal epilepsy research data that includes electrophysiological data in EDF format, imaging data in DICOM format, and genomic data as well. Data flow consists of the following main steps: (1) patient demographics are entered, and unique CSR study identifiers are generated; EMU reports are generated using NHash identifier, (2) larger files (eg, EEG, imaging) are generated from patient care and named using NHash identifier, (3) patient data (demographics, history, medication, diagnosis) and EMU reports are de-identified and exported to the CSR central data repository (IDAC), (4) EEG, imaging, and other larger files are also de-identified and exported to IDAC, and (5) exported data from all CSR clinical sites are aggregated into a single repository (MEDCIS) for searching, querying, and sharing.  

## Results

### NHash Identifier Generation in Ontology-Driven Patient Information Capture

**Figure 5:**
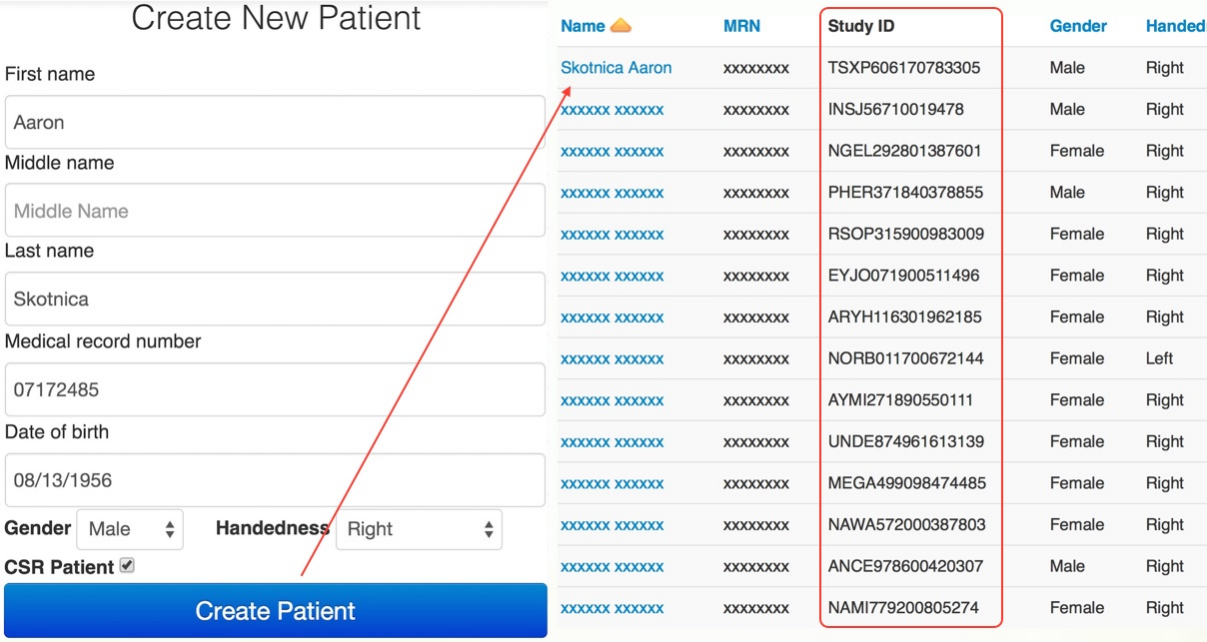
Screenshot of OPIC interface generating and displaying NHash identifiers.

An NHash identifier is generated at patient enrollment phase using the algorithm in Figure 3 and managed through the Patient Demographics module of OPIC. Figure 5 shows the patient registration interface using our fictional study participant, Aaron Skotnica (left part of Figure 5). For CSR, we use DOB 08/13/1956 and MRN 07172485, among all captured data elements, to generate the unique NHash identifier TSXP606170783305 (top right, Figure 5). Ten additional actual NHash identifiers are displayed inside the area marked by a red rectangle.

### Linking Multimodal Data in MEDCIS

**Figure 6:**
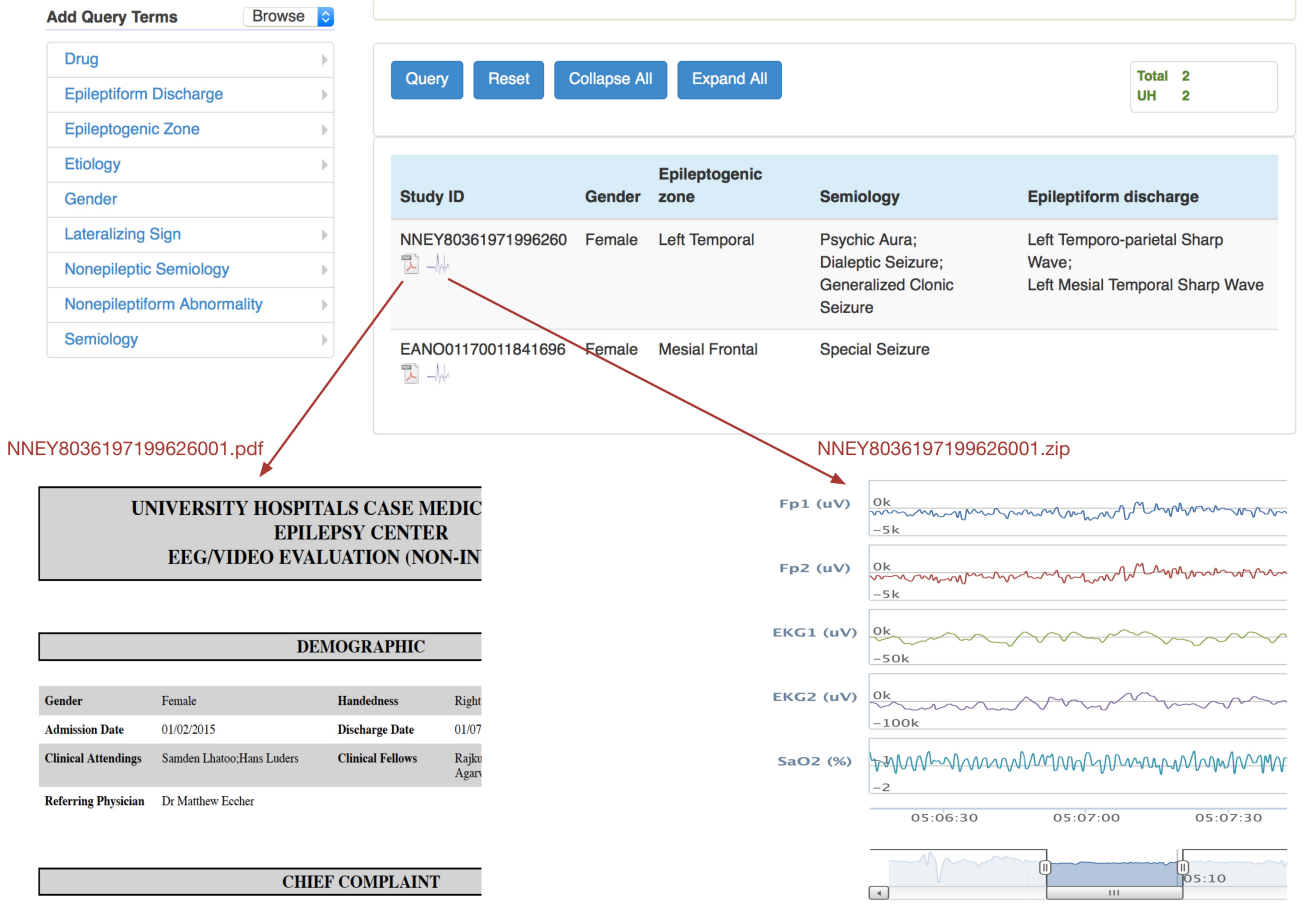
Screenshot of the MEDCIS query interface for searching, querying, and sharing linked multimodal clinical data.

MEDCIS is implemented using agile Web development with the Web application framework, Ruby on Rails. MEDCIS has been deployed [10] for searching, querying, and sharing the CSR multimodal clinical data linked by NHash identifiers. Figure 6 shows a screenshot of the MEDCIS query interface for CSR. The top part is the MEDCIS ontology-driven query interface [7] following the VISAGE design [11]. The results of the query include key characteristics of seizures, as well as links to related multimodal clinical data, displayed below for discharge summary report in PDF and EEG signals in EDF format. The query result returns the number of study participant reports satisfying the query criteria, as well as detailed participant information including NHash identifier, gender, epileptogenic zone, seizure semiology, and epileptiform discharge. Moreover, MEDCIS provides two hyperlinks (below the NHash identifier) to the de-identified discharge summary report (in PDF) and electrophysiological signal data (in EDF) of the study participant, which can be downloaded or viewed. As indicated in the lower part of Figure 6, NHash identifiers are directly used for linking with associated multimodal clinical data for immediate access.

### Deployment

The CSR Institutional Review Board and Data Use Agreements were approved by nine clinical sites involved in the recruitment of patients for the SUDEP study and the deployment of the MEDCIS tools including OPIC, using a common template accessible by participating institutions [12]. Secure Sockets Layer (SSL) has been used for all Web accesses of CSR resources. SSL provides a secure connection between Internet browsers and websites, allowing the transmission of data in encrypted form in transit. Access to the CSR data resource is password-protected, with built-in administrative auditing tools for tracking resource usage activities.

De-identified data were ported to the center repository according to CSR data flow (shown in Figure 4) in two ways: (1) a regular structured database dump to the MEDCIS Web server for cohort search, and (2) secure file transfer protocol (SFTP) to upload larger multimodal files from within hospital firewalls to the data repository. Since the CSR project inception in October 2014, 341 patients have been enrolled (including those enrolled in the pilot phase of the study), and 7TB of data have been moved into the CSR data repository for sharing.

###  Evaluation

One of the key trade-offs in designing NHash study identifiers is the need for exclusion of any sensitive pieces of data, versus the desire to be able to easily check the validity of a study identifier (even without using the protected codebook). In one extreme of the NHash identifier design, one could use random numbers only, without any of the N-gram hashes (ie, using 0-grams for all components of the identifier). The other extreme is the use of longer N-grams, which would not be desirable because more PHI-related information bits would be revealed.

We believe that a balance could be reached between the two extremes with NHash because our general design of the NHash study identifier is flexible in a number of ways. One is that it allows a variety of study participant information to be included (or not included) for the hash functions. The second is that the sizes of the N-grams (the N) involved are parameters that can be adjusted from study to study, based on specific needs.

**Table 2:** Analysis of probability for collision.

N-grams	Inverse probability (lower bound)	Inverse probability (upper bound)
Name	10^4^	26^4^
MRN	9^4^	10^4^
DOB	10^2^	10^2^
Random number	10^6^	10^6^
Total	6.6 × 10^15^	4.6 × 10^17^
EC for 10^6^ records	0.000076	1.09× 10^-6^
EC for 10^7^ records	0.0076	1.09× 10^-4^
EC for 10^8^ records	0.76	0.0109

The shift operation in the shift-cipher does not change the inverse probability of the ID generation, thus the expected collision is the same with or without the encryption in the last step of NHash. For CSR, we used a 4-gram from name (name component), a 4-gram of medical record number (MRN component), and a 2-gram from date of birth (DOB component), together with a random number between 0 and 1,000,000 to generate the NHash identifier.

One of the basic desired properties of study identifiers is uniqueness: each study participant should have an identifier that is distinct from all other (current and future) identifiers for a study. This uniqueness property is sometimes called collision-free, or free of false identity [3]. Given a hash function with inverse probability of N, the expected number of collisions (EC) after I insertions is EC=I - N + N(1 - 1/N)^I^.

To see how this formula is derived, let EE be the expected number of empty slots. Then EC=I – (N – EE) since N – EE is the number of occupied slots. For insertion, the probability that a specific slot is occupied is 1/N. Thus the probability of a slot not being occupied by this element is (1 – 1/N). After I insertions, the probability of a slot remaining unoccupied is (1 – 1/N)^I^. With *N* slots, the expected number of empty slots is N(1 – 1/N)^I^.

For example, if the inverse probability of the hash function is 6.6 × 10^15^(the lower bound inverse probability in Table 2), after 1,000,000 records, the expected number of collisions is 1,000,000 – 6.6 × 10^15^ + 6.6 × 10^15^(1 – 1/6.6 × 10^15^)^1,000,000^. This equals 0.000076. In Table 2, we used the lengths of the overwhelming majority of surnames (5) and given names (5) [13,14]. The MRN number is assumed to be 8 digits long, with the possibility of the leading digit being 0. Even though the entire DOB has 8 digits, the year range has only 2 effective digits that can vary in the full range for the current population. Thus, we underestimate the possibility of 2-grams to be 10^2^ only. The expected number of collisions also implies that there is only 0.0076% chance for generating a collision after inserting 1,000,000 records. Therefore, for CSR, the probability of collision or false-identity is extremely low, and we have so far encountered no collisions at all in its deployment within a local instance.

### Simulation

To further validate the effectiveness of NHash, we performed experiments using a large synthesized dataset comparing NHash with random strings. There are two goals using random string methods in our study: (1) random strings are good benchmark methods for generating unique strings, and (2) validating our experiment design by comparing the expected collision and the number of collisions from the experiment.

It should be noted that the goal of NHash is not to outperform the random string methods in terms of minimizing collision but to provide a hash function with comparable expected collision and the desirable features presented in previous sections.

The synthesized datasets are generated using random names, MRN, and DOB. The names are generated based on the list of top 5000 surnames and 2500 first names in the United States. The frequency of the names is factored in our simulation. The MRN is generated as an 8-digit random number, and the date of birth is a random date from 1910 to 2015. We generated five datasets with 100 million records each.

Table 3 lists the number of collisions using different hash functions. The expected number of collisions is very close to the average number of collisions for all hash functions, and the derivation from the average is low. From Table 3, the number of collisions in NHash-15 is very close to Random-13 and NHash-16 is very close to Random-14, thus clearly indicating the effectiveness of NHash.

**Table 3:** Number of collisions on synthesized dataset of 100 million (n in Random-n indicates the length of the random string; NHash-15 uses random numbers of length 5 and NHash-16 uses random numbers of length 6).

	Run 1	Run 2	Run 3	Run 4	Run 5	Average	EC
Random -11	1088	1088	1103	1131	1134	1108.8	1086.95
Random-12	110	106	128	123	108	115	108.70
Random-13	9	13	16	8	1	11.2	10.87
Random-14	0	0	0	2	0	0.4	1.09
NHash-15	6	7	5	1	4	4.6	7.58
NHash-16	0	0	1	0	0	0.2	0.76

## Discussion

### Principal Findings

NHash is a generalizable mechanism for generating study identifiers in a multicenter research setting. In the first phase of generating an intermediate string, the size of randomized N-gram, that is, the number N, is adjustable. In the second phase of further encryption, we used the Cantor paring function for shift-cipher. Other functions and encryption techniques can also be utilized in this phase. The encryption phase provides the mechanism to prevent the NHash scheme from dictionary attacks.

In related work, Johnson [3] proposed a centralized method for generating global unique identifiers to link collections of research data and specimens for Autism spectrum disorder. There are two ways our approach differs. One is that our NHash identifiers are generated distributively, rather than using a centralized Web service. This has advantages in reduced administrative overhead and simplified workflow (since everything is local). Another advantage is that NHash does not require the input of identifiable information into a centralized Web service, which can be perceived as a risk by local sites. The disadvantage is the possibility for false-split: if a study participant is enrolled in two or more sites (on rare occasion), then the participant would be treated as distinct.

The second distinction is in the use of a nondeterministic random number. In Johnson’s work [3], the central random identifier generated is the global unique identifier, with a common prefix. For NHash, the random number is part of the identifier but it is also used for generating N-gram hashes that are part of the identifier. This feature makes NHash validatable and much more robust to manual errors compared with typical hash functions. One simple typical method is to assign study participants with sequential unique identifiers according to the creation time. For example, 00001 is assigned for the first study participant, 00002 is assigned for the second study participant, and so forth. Such methods are prone to manual errors, for instance, the study identifier of the first study participant is incorrectly typed as 00002 instead of 00001. In this case, researchers will not be able to tell if the identifier is correct since the study identifier, 00002, by itself represents a valid study participant. However, NHash can easily detect the correctness of the identifier. Taking the same fictional study participant, Aaron Skotnica, as an example. His study identifier is TSXP606170783305. If it is typed as TSXP606170783306 by mistake, NHash is able to detect that it is an invalid study identifier. Based on the algorithm in Figure 3, we know that the postfix 783306 is the random number and that the study identifier would be SXPT06130208783306.

### Limitations

The first limitation concerns managing identifier collision in a decentralized setting. Our implementation involves local collision checking (Figure 2) so that all possible identifier collisions within a site are already avoided. We do not have an automated method for checking identifier collision across sites. Even though the probability of identifier collision is extremely small, a process needs to be in place to address it when it happens. We address cross-site identifier collision at data merging stage (see Figure 4). If and when a new batch of data involves an identifier that already exists in the central repository, the local site will regenerate an identifier that is distinct from existing ones. All associated data will be renamed accordingly. However, if the same patient appears at two participating sites, the proposed identifier generation mechanism might not be able to find out that they are the same person.

The second limitation relates to the required data fields to be used for generating NHash. If data for any of the fields are missing or inaccurate, it could create undesirable results. We address this limitation in CSR by reviewing the accuracy and completeness of the patient record before the NHash identifier is generated.

###  Conclusions

This paper introduces a novel method, NHash, for generating unique study identifiers in a distributed and validatable fashion, in multicenter research involving prospectively collected and de-identified study data. NHash has been deployed for linking multimodal, multicenter data for the Center for SUDEP Research, a National Institute for Neurological Disorders and Stroke–funded Center Without Walls for Collaborative Research in the Epilepsies. Since the official launching of CSR in December 2014, 341 study subjects have enrolled in the SUDEP study, with nearly 7TB of EEG signals linked using identifiers generated using NHash. NHash provides a de-centralized, lightweight study identifier algorithm that offers choices for balancing the trade-offs among the competing requirements of freedom from false-identity and split-identity, and minimal risk for attacks.

## Abbreviations

CSR: Center for SUDEP Research
HIPPA: Privacy Rule of the Health Insurance Portability and Accountability Act
IDAC: Informatics and Data Analytics Core
MEDCIS: Multi-Modality Epilepsy Data Capture and Integration System
NHash: Randomized N-gram Hashing
NINDS: National Institute for Neurological Disorders and Stroke
OPIC: Ontology-Driven Patient Information Capture
PHI: protected health information
SUDEP: sudden unexpected death in epilepsy patients

## References

[1] U.S. Department of Health & Human Services.

[2] Johns Hopkins Bloomberg School of Public Health.

[3] Johnson SB, Whitney G, McAuliffe M, Wang H, McCreedy E, Rozenblit L, Evans CC (2010). Using global unique identifiers to link autism collections. J Am Med Inform Assoc.

[4] Epilepsy Foundation.

[5] Boon P, Vonck K, De HV, Van DA, Goethals M, Goossens L, Van ZM, De ST, Dewaele I, Achten R, Wadman W, Dewaele F, Caemaert J, Van RD (2007). Deep brain stimulation in patients with refractory temporal lobe epilepsy. Epilepsia.

[6] Fisher RS (1993). Emerging antiepileptic drugs. Neurology.

[7] Zhang G-Q, Cui L, Lhatoo S, Schuele S, Sahoo S (2014). MEDCIS: Multi-Modality Epilepsy Data Capture and Integration System. AMIA Annu Symp Proc.

[8] Sahoo SS, Lhatoo S, Gupta D, Cui L, Zhao M, Jayapandian C, Bozorgi A, Zhang G (2014). Epilepsy and seizure ontology: towards an epilepsy informatics infrastructure for clinical research and patient care. J Am Med Inform Assoc.

[9] Sahoo SS, Zhao M, Luo L, Bozorgi A, Gupta D, Lhatoo SD, Zhang GQ (2012). OPIC: Ontology-driven Patient Information Capturing system for epilepsy. AMIA Annu Symp Proc.

[10] MEDCIS Cross Cohort Query Interface.

[11] Zhang G-Q, Siegler T, Saxman P, Sandberg N, Mueller R, Johnson N, Hunscher D, Arabandi S (2010). VISAGE: A Query Interface for Clinical Research. AMIA Jt Summits Transl Sci Proc.

[12] The Center for SUDEP Research (CSR).

[13] The U.S. Census Bureau.

[14] Official Social Security Website.

